# Validation of the Brazilian-Portuguese Version of a Short Questionnaire to Assess Knowledge in Cardiovascular Disease Patients (CADE-Q SV)

**DOI:** 10.5935/abc.20180169

**Published:** 2018-12

**Authors:** Gabriela Lima de Melo Ghisi, Gabriela S. S. Chaves, Jessica Blanco Loures, Gabriela Moreira Bonfim, Raquel Britto

**Affiliations:** 1 Toronto Rehabilitation Institute, University Health Network, Toronto - Canada; 2 Universidade Federal de Minas Gerais (UFMG), Belo Horizonte, MG - Brazil

**Keywords:** Cardiovascular Diseases, Coronary Artery Disease, Surveys and Questionnaires, Patient Education as Topic, Knowledge, Educational Status

## Abstract

**Background:**

Patient education is an essential part of cardiovascular patients' care targeting self-management behavior to reduce risk factors and subsequent events. Herein, a short and reliable tool to assess patients' knowledge in Brazil is warranted.

**Objectives:**

To translate, culturally-adapt and psychometrically validate the Portuguese version of the Coronary Artery Disease Education Questionnaire Short Version (CADE-Q SV).

**Methods:**

The Portuguese CADE-Q SV - translated and culturally-adapted - was reviewed by five bilingual experts in cardiovascular disease. This version was then pre-tested in 21 patients, and clarity of items was checked using a Likert-type scale ranging from 1 = not clear to 10 = very clear. It was then psychometrically tested in 200 cardiovascular patients (41%women; mean age = 58.4 ± 11.6 years old). The internal consistency was assessed using Kuder-Richardson-20 (KR-20) and Cronbach's alpha, test-retest reliability through intraclass correlation coefficient (ICC), factor structure using confirmatory factor analysis, and construct validity regarding educational level, family income, and time of diagnosis.

**Results:**

All questions were considered clear by patients (clarity range:7.8-9.6). KR-20 was 0.70. All ICC values were > 0.70. Factor analysis revealed 6 factors, all internally consistent. Construct validity was supported by significant differences in total scores by educational level and family income (p < 0.001). The overall mean was 13.08 ± 2.61. The area with the highest knowledge was risk factors and the lowest was psychosocial risk.

**Conclusions:**

The Portuguese CADE-SV was demonstrated to have good validity and reliability. This tool can be applicable in clinical and research settings, assessing cardiovascular patients' knowledge as part of an education programming.

## Introduction

Cardiovascular diseases (CVDs) are among the leading burdens of disease and disability worldwide,^1^particularly in low and middle-income countries (LMICs) such as Brazil.^2^ Cardiac rehabilitation (CR) is an outpatient secondary prevention care model designed to mitigate this burden.^3^ Indeed, participation in CR has been shown to reduce morbidity and mortality by 20%, in a cost-effective manner.^4-7^ Improved risk factor control, psychosocial well-being, and health behaviors are also shown in LMICs with CR participation.^8^ However, there are incredibly few studies in this setting showing the long-term success of CR, which rests in part on the patient's ability to maintain health behaviors, including participation in regular physical activity after the end of the program.^9,10^

Patient education is an essential part of the rehabilitation of CAD patients targeting self-management behavior to reduce risk factors and subsequent cardiac events.^11^The American and Canadian Cardiovascular Societies include patient education as a quality indicator of CR,^12,13^ and this component is also recommended in the delivery of CR in LMICs.^14^Indeed, meta-analyses of education for cardiovascular patients suggest it is associated with improvements in self-management behaviors,^9-11,15^ health-related quality of life,^16^ decreases in healthcare costs,^16^and recurrence of acute events.^15^

In this context, the Coronary Artery Disease Education Questionnaire (CADE-Q) was previously developed and psychometrically validated as a valid and reliable tool to inform Brazilian healthcare providers oft what their cardiovascular patients know about their condition.^17^It was later validated to English.^18^ It has also been used in several studies, including randomized controlled trials.^19^ Although both versions demonstrated good reliability and validity, CADE-Q presented lack of detailed assessment of all core components of cardiac rehabilitation, such as nutrition and psychosocial risk. Therefore, a second version (CADE-Q II) was developed and validated in English.^20^ However, both tools take around 20 minutes to be completed, and there was a need for a short and quick instrument to more easily assess CR patients' knowledge in clinical practice. This tool was validated in English and it is called CADE-Q SV.^21^The aim of this study was to translate, culturally-adapt and psychometrically validate a Brazilian-Portuguese version of CADE-Q SV.

## Methods

### Design and Procedures

The design of this study consisted of a series of cross-sectional, observational studies. Data was collected between September 2017 and February 2018.

First, the translation and cultural adaptation was performed. This process followed strict norms approved by the author and co-authors and was based on the protocol proposed by Guillemin et al:^22^ (1) initial translation, (2) back-translation, (3) committee review of those translations and back-translations, and (4) pre-testing for equivalence using bilingual individuals. The initial translation was performed by an independent translator, aware of the objectives and concepts underlying the study and sought to detect ambiguities and unexpected meanings in the original items. The back-translation was performed by a second translator, blinded to the initial objectives of the study and the original version. All versions were reviewed by a committee of three bilingual experts. This version was then pre-tested in 20 patients and clarity of items was checked. To assess clarity, patients were asked to rate each item on a Likert-type scale ranging from 1 (not clear) to 10 (very clear). Results were used to refine the Brazilian-Portuguese version of CADE-Q SV.

Second, a psychometric validation was performed. The refined tool was administered to a larger sample of current cardiovascular ambulatory patients from a public hospital in Belo Horizonte, Minas Gerais. The instrument was applied through monitored self-administration (i.e. researchers maintained a neutral stance during the administration, answering questions about the research and encouraging participants to answer all questions). The questionnaire was re-administered one month after the first application in 21 randomly selected participants to assess test-retest reliability. Data were collected between June and November 2017.

### Participants

For the psychometric validation, a convenience sample of 200 ambulatory cardiovascular patients was recruited. The sample size calculation for this analysis was based on Hair &Anderson's^23^recommendation of 10 subjects per item. Since CADE-Q SV has 20 items, a sample size of 200 is considered valid. The inclusion criteria were the following: confirmed cardiac diagnosis or multiple cardiovascular risk factors. The exclusion criteria were the following: younger than 18 years old, illiterate, any significant visual, cognitive or mental impairment which precludes the participant's ability to answer the questionnaire.

CR participants were characterized according to gender, age, educational level, family income, comorbidities, clinical risk factors, and history and duration of participation in CR. The participant's clinical characteristics were obtained from the medical chart, and socio-demographic characteristics were self-reported.

### Measure: The CADE-Q SV scale

CADE-Q SV assesses cardiovascular patients' knowledge about their condition. It was designed to be a true/false/I don't know questionnaire, with 20 items, four in each domain as follows: medical condition, risk factors, exercise, nutrition, and psychosocial risk. Each correct answer equals to one point; therefore, the maximum score possible is 20 overall, four by domain, and one per item. The tool has been developed in English and psychometrically tested in Canadian CR participants.^21^This tool can be used to tailor any type of educational intervention addressed to cardiovascular patients, not only in CR programs.

### Statistical analysis

SPSS Version 24.0 was used.^24^ The level of significance for all tests was set at 0.05. Psychometric properties were tested as per the Consensus-based Standards for the selection of health Measurement Instruments (COSMIN) taxonomy.^25^First, internal consistency was assessed by the Kuder-Richardson-20 (KR-20) overall, and by Cronbach's alpha of each factor (based on factor structure, described below). For this analysis, values equal to, or higher than 0.70 were considered acceptable,^23^ reflecting the internal correlation between items of the same area.

Second, factor structure was assessed using confirmatory factor analysis. The main component method for factor extraction was used with consideration being given only to those with eigen values > 1.0. After the selection of the factors, a correlation matrix was generated, whereby the associations between items and factors were observed through factor loadings greater than 0.40 on only one factor.^23^ The varimax method with Kaiser normalization was used to interpret the matrix.^26^

Third, test-retest reliability was assessed using intraclass correlation coefficient (ICC). ICC values lower than 0.70^27^ were considered bad items. Finally, criterion validity was also assessed by comparing CADE-Q SV total scores with the participant's level of education, family monthly income and time of diagnosis, using independent sample t-tests and Pearson's correlation. Item completion rates were also described.

A descriptive analysis of the Portuguese CADE-Q SV was performed. A mean total score was computed to reflect total knowledge. Independent sample t-tests, one-way analysis of variance, and chi-square tests were used as appropriate to assess differences in total knowledge based on patient's socio-demographic and clinical characteristics. Continuous variables were all normally distributed (confirmed by Kolmogorov-Smirnov test) and were reported with mean and standard deviations. Categorical variables were reported by absolute numbers, percentages and, when applicable, confidence intervals.

## Results

### Participants' characteristics

The characteristics of participants from the psychometric validation are described in Table 1. Overall, 200 cardiovascular ambulatory patients completed the Portuguese version of CADE-Q SV, of which 118 (59.0%) were male, and the mean age was 58.4 ± 11.6 years old.

**Table 1:** Sociodemographic/Clinical Characteristics of the Participants and total scores and differences among subgroups (n = 200)

Characteristic		CADE-Q SV Total Score		
**Sociodemographic**			**(mean ± SD)**	**p**
Age, years (mean ± SD)		58.4 ± 11.6	-	-
Age dichotomous n (%)	Less than 65 years old	132 (66.0)	13.6 ± 2.4	0.001^†^
	65 years old or older	68 (34.0)	12.2 ± 2.8	
Sex n (%)	Male	118 (59.0)	13.3 ± 2.5	0.23
	Female	82 (41.0)	12.8 ± 2.8	
Educational level n (%)	Never went to school	8 (4.0)	12.0 ± 3.2	< 0.001^‡^
	Less than High School	128 (64.0)	12.5 ± 2.5	
	High School	54 (27.0)	14.1 ± 2.4	
	University	8 (4.0)	15.5 ± 1.2	
	Post-graduation	2 (1.0)	17.0 ± 0.0	
Monthly family income n (%)	No income	15 (7.5)	12.6 ± 1.8	0.04*
	Less than 1 minimum salary	98 (49.0)	12.8 ± 2.7	
	Between 1 and 3 minimum salaries	69 (34.5)	13.2 ± 2.6	
	Between 4 and 5 minimum salaries	12 (6.0)	14.2 ± 2.0	
	6 or more minimum salaries	4 (2.0)	16.0 ± 2.0	
**Clinical**				
Acute Cardiac Event n (%)	Acute Myocardial Infarction	113 (56.5)	13.5 ± 2.5	0.03*
Comorbidities n (%)	Hypertension	179 (89.5)	13.1 ± 2.6	0.91
	Dyslipidemia	138 (69.0)	13.1 ± 2.5	0.81
	Stress	71 (35.5)	13.1 ± 2.5	0.81
	Peripheral Obstructive Arterial Disease	54 (27.0)	12.5 ± 3.0	0.06
	Diabetes Type II	53 (26.5)	13.4 ± 2.2	0.36
	Arrhythmia	51 (25.5)	12.4 ± 2.4	0.04*
	Stable Apnea	42 (21.0)	12.9 ± 2.9	0.58
	Depression	41 (20.5)	12.8 ± 2.4	0.37
	Obesity	40 (20.0)	13.3 ± 2.6	0.65
	Unstable Angina	37 (18.5)	12.7 ± 2.2	0.55
	Smoking	19 (9.5)	14.4 ± 2.2	0.08
	Alcoholic behaviour	6 (3.0	14.5 ± 1.9	0.18
**Time from diagnosis**				
Time from diagnosis, years (mean±SD)		8.6 ± 9.1	-	-
Time from diagnosis, n (%)	Less than 1 year	50 (25.0)	13.6 ± 2.4	0.23
	Between 1 and 5 years	44 (22.0)	12.6 ± 2.8	
	Between 6 and 10 years	23 (11.5)	12.9 ± 2.6	
	Between 11 and 15 years	25 (12.5)	13.9 ± 2.2	
	More than 15 years	38 (19.0)	12.6 ± 2.6	

SD-standard deviation; Significant differences between groups:

(*) p < 0.05,

† p < 0.01,

‡ p < 0.001.

Note: Income shown in Brazilian minimum salaries. One minimum salary corresponds to BRL$ 954,00 or USD$ 292.95.

### Translation, cultural adaptation and pre-testing

During the process of translation and cultural adaptation, it was observed that one item needed to be adapted to be used in the Brazilian context *(item 11). Previously, this item had names of statin medications popular in North America, and since the tool was used in different countries it was adapted to read “ ‘*Statin' medications (such as atorvastatin and simvastatin) limit how much cholesterol your body absorbs from food”. Based on the feedback received from the experts we have included two examples of popular medications used in Brazil. No other adaptations were made. Table 2 displays all items of the Portuguese version of CADE-Q SV.

**Table 2:** Clarity (n = 21), means and Standard Deviations of CADE-Q SV scores per item, item completion rates (n = 200), ICC (n = 20), and Mean Scores per area

Area	Item	Clarity* Mean ± SD	Score Mean ± SD	Item completion rates (%)	ICC	Mean Score Per area
1 - Medical	1. Heart disease only happens in older people who smoke or have high cholesterol.	8.5 ± 1.9	0.73 ± 0.45	98.5	0.75	2.38 ± 0.76
	3. "Angina" is chest pain or discomfort in your arm, back or neck.	8.1 ± 3.0	0.75 ± 0.44	98.5	0.71	
	6. Medications such as aspirin (ASA) help prevent blood clots from forming.	8.5 ± 2.6	0.86 ± 0.35	98.5	0.70	
	11. "Statin" medications (such as atorvastatin and simvastatin) limit how much cholesterol your body absorbs from food.^†^	8.8 ± 1.8	0.05 ± 0.21	98.5	0.72	
2 - Risk Factors	2. Lifestyle changes such as healthy eating can lower your chances of developing heart disease.	9.1 ± 1.9	0.89 ± 0.32	98.0	0.80	2.95 ± 0.88
	12. To help control your blood pressure, eat less salt and exercise regularly.	9.5 ± 0.8	0.97 ± 0.16	98.5	0.83	
	16. To control cholesterol, become a vegetarian and avoid eating eggs.	8.7 ± 1.5	0.51 ± 0.50	98.5	0.77	
	18. You cannot prevent diabetes with exercise and healthy eating.	8.7 ± 2.1	0.58 ± 0.49	98.5	0.85	
3 - Exercise	4. Resistance training (lifting weights or using elastic bands) can strengthen your muscles and help lower your blood sugar.	8.0 ± 2.5	0.63 ± 0.48	98.5	0.72	2.69 ± 1.01
	8. A warm-up before exercising raises your heart rate and lowers your chance of getting angina.	8.8 ± 1.6	0.63 ± 0.48	98.5	0.70	
	13. If you get chest discomfort while walking, speed up to see if it goes away.	9.0 ± 1.6	0.86 ± 0.35	98.5	0.79	
	17. You are exercising at the right level when your heart rate is in the target zone and you can still talk comfortably.	8.4 ± 2.4	0.57 ± 0.50	98.5	0.80	
4 - Diet	5. Eating more meat and dairy products is a good way to add more fiber to your diet.	8.1 ± 2.2	0.47 ± 0.50	98.0	0.72	2.09 ± 0.84
	9. Prepared or processed foods, such as canned soup and bacon, usually have a lot of salt (sodium).	8.8 ± 2.1	0.90 ± 0.30	98.5	0.98	
	14. Trans fat is an unhealthy type of fat that is often found in baked or fried foods.	7.8 ± 2.9	0.78 ± 0.41	98.5	0.74	
	20. To help lower your blood pressure, eat healthy foods more often, such as vegetables, fruits, and whole grains.	9.6 ± 0.9	0.94 ± 0.24	98.5	0.94	
5 - Psychosocial Risk	7. The only effective way to manage stress is to avoid people who cause unpleasant feelings.	8.2 ± 3.0	0.35 ± 0.48	98.5	0.77	1.97 ± 0.70
	10. Depression is common after a heart attack and increases the chance of having another heart attack.	8.5 ± 2.2	0.63 ± 0.48	98.0	0.78	
	15. Sleep apnea (pauses in breathing during sleep) can increase your chance of having another heart attack.	8.1 ± 2.7	0.05 ± 0.21	98.5	0.77	
	19. Stress increases your chance of having a heart attack as much as high blood pressure and diabetes.	9.1 ± 1.4	0.94 ± 0.23	98.5	0.72	
Total		8.6 ± 3.2	13.08 ± 2.61	-	-	-

SD-standard deviation; ICC-intraclass correlation coefficient;

* Clarity was assessed using a Likert-type scale ranging from 1 = not clear to 10 = very clear;

† item culturally adapted. Note: maximum score for item is 1 and for areas is 5.

Table 2 also presents the clarity of items graded by 21 cardiovascular patients as part of the pre-testing using a Likert-type scale ranging from 1 (not clear) to 10 (very clear). Clarity of items ranged from 7.8 to 9.6, and overall clarity of the tool was 8.6 ± 3.2, which shows the Portuguese version of CADE-Q SV was clear to patients.

### Psychometric validation

The internal consistency of the entire sample was assessed by KR-20(0.70). Regarding factor analysis, results from the Kaiser-Meyer-Olkin index (KMO = 0.78) and Bartlett's Sphericity tests (*X^2^* = 490.481, p < 0.001) indicated that the data were suitable for factor analysis. Six factors were extracted, representing 59.0% of the total variance. All factors were reliable (Cronbach's alpha ranged from 0.70-0.81). These factors were called: medical, risk factors, exercise, diet, psychosocial risk, and specific cases. Table 3 shows the factor loadings for each item based on loadings greater than 0.30 on only one factor.

**Table 3:** Factor loadings from confirmatory factor analysis

Items	Factor 1: Specific cases	Factor 2: Exercise	Factor 3: Diet	Factor 4: Medical	Factor 5: Risk factors	Factor 6: Psychosocial risk
10. Depression is common after a heart attack and increases the chance of having another heart attack.	0.47					
11. "Statin" medications (such as atorvastatin and simvastatin) limit how much cholesterol your body absorbs from food.	0.39					
15. Sleep apnea (pauses in breathing during sleep) can increase your chance of having another heart attack.	0.39					
18. You cannot prevent diabetes with exercise and healthy eating.	0.31					
4. Resistance training (lifting weights or using elastic bands) can strengthen your muscles and help lower your blood sugar.		0.33				
8. A warm-up before exercising raises your heart rate and lowers your chance of getting angina.		0.46				
13. If you get chest discomfort while walking, speed up to see if it goes away.		0.48				
17. You are exercising at the right level when your heart rate is in the target zone and you can still talk comfortably.		0.47				
5. Eating more meat and dairy products is a good way to add more fiber to your diet.			0.45			
9. Prepared or processed foods, such as canned soup and bacon, usually have a lot of salt (sodium).			0.46			
14. Trans fat is an unhealthy type of fat that is often found in baked or fried foods.			0.56			
20. To help lower your blood pressure, eat healthy foods more often, such as vegetables, fruits, and whole grains.			0.38			
1. Heart disease only happens in older people who smoke or have high cholesterol.				0.52		
3. "Angina" is chest pain or discomfort in your arm, back or neck.				0.39		
6. Medications such as aspirin (ASA) help prevent blood clots from forming.				0.44		
2. Lifestyle changes such as healthy eating can lower your chances of developing heart disease.					0.30	
12. To help control your blood pressure, eat less salt and exercise regularly.					0.56	
16. To control cholesterol, become a vegetarian and avoid eating eggs.					0.34	
7. The only effective way to manage stress is to avoid people who cause unpleasant feelings.						0.52
19. Stress increases your chance of having a heart attack as much as high blood pressure and diabetes.						0.32
Variance	17.3	11.1	9.4	8.2	6.9	6.6
Eigenvalues	3.3	1.6	1.5	1.2	1.2	1.1
Cronbach's Alpha	0.73	0.81	0.79	0.70	0.71	0.70

(*) item culturally adapted.

The test-retest reliability was evaluated through the ICC for each item, and the ICCs for all items meet the minimum recommended standard. In regard to construct validity, CADE-Q SV total scores were compared by participant's level of education, family monthly income and time of diagnosis. As shown in Table 1, patients with lower educational level had significantly higher needs than those with higher education (p < 0.001), and participants with no income or less than 1 minimum salary had lower knowledge than participants that earn 4 minimum salaries per month or higher (p < 0.05). No differences were found regarding time of diagnosis.

### Cardiovascular patients' knowledge about their condition

Table 2 displays the means and standard deviations of each CADE-Q SV item, as well as total scores per area. Items with the highest scores (i.e., with the highest number of correct answers) were the following: “to help control your blood pressure, eat less salt and exercise regularly”, “stress increases your chance of having a heart attack as much as high blood pressure and diabetes”, and “to help lower your blood pressure, eat healthy foods more often, such as vegetables, fruits, and whole grains”. Items with the lowest knowledge (i.e., items with the lowest scores) were the following: “ ‘statin' medications (such as atorvastatin and simvastatin) limit how much cholesterol your body absorbs from food”, “sleep apnea (pauses in breathing during sleep) can increase your chance of having another heart attack”, and “the only effective way to manage stress is to avoid people who cause unpleasant feelings”. The area with the highest knowledge was risk factors and the one with the lowest was psychosocial risk. Patients spend around 10 minutes to complete the tool.

Table 1 presents the total score per participant's characteristics. As displayed, patients that had a myocardial infarction or have arrhythmia had significantly higher knowledge than their counterparts (p < 0.05). In addition, younger participants (i.e. less than 65 years old) had significantly higher knowledge than participants who were 65 years old or older.

## Discussion

Education is a core component of CR and cardiac care, and is necessary to promote patient's understanding of secondary prevention strategies and adherence to these strategies. Herein, a short and reliable tool to assess cardiovascular patients' knowledge - called CADE-Q SV - has been translated, culturally adapted, and psychometrically validated through a rigorous process. Internal consistency, test-retest reliability, criterion validity, and factor structure were all established, and demonstrate the utility of this tool.

Results of this study were consistent with those presented in the original validation,^21^ particularly in relation to criterion validity (correlation to educational level) and all areas being considered internally consistent (α > 0.70). In this validation, there are 6 factors, even thoughthe tool has 5 areas. The new factor was called “specific cases” and included questions related to comorbidities and specific diagnosis that may not be relevant to all cardiovascular patients (e.g., diabetes and sleep apnea). Adult patients learn based on their personal needs and when the information is not relevant to them they may not have interest to learn about it.^28,29^ Therefore, these items were combined in one factor and in future studies with the tool, researchers should flag these items and see if cardiovascular patients with or without these comorbidities will have the same knowledge.

The overall mean, as well as the means of the areas, were low, reinforcing the need for educational strategies to teach cardiovascular patients, which have been reinforced in publications about strategies to treat these patients in low-and middle-income countries.^14^ Thus, the areas with higher knowledge in this study (risk factors) were different from the areas identified in the original validation (exercise and diet).^21^ This result was expected since in this study we have administered the survey in ambulatory cardiovascular patients, while the original study was with CR patients.

Future research is needed to further establish the psychometric properties of the Portuguese version of CADE-Q SV. First, in relation to the potential strategies to educate cardiovascular patients, it should be determined whether the scale is sensitive to change (i.e., responsiveness), such as after CR or educational programs. Second, there are other measurement properties of the scale that require assessment, such as criterion validity. Moreover, test-retest reliability was performed in 20 patients, and the literature points that the minimum number should be 50.^27^ Third, the type of sample and the fact that participants were recruited from only one site also limits this study. Therefore, the Portuguese CADE-Q SV should be administered in other health programs and Brazilian states, to ensure it is appropriate and performs well in more general settings. Finally, future research is needed to assess whether the scale is sensitive to change, such as following participation in CR, or to test implementation of new education materials. Second, whether CADE-Q SV is a valuable and valid tool to identify knowledge differences in CR patients should be explored.^30^ For this study patients did not receive any feedback regarding their knowledge; however, we encourage clinicians and researchers to provide this to patients.

## Conclusions

In conclusion, the Portuguese version of CADE-Q SV proved to have strong psychometric properties, providing preliminary evidence of its validity and reliability to assess cardiovascular patients' knowledge in Brazil. It is hoped that this tool can support healthcare providers and CR programs to evaluate their patients' knowledge in clinical practice and promote greater provision of educational strategies.

The use of the Portuguese version of CADE-Q SV for clinical and research purposes will be free of charge, and all information - including the tool - is available online at https://cadeq.wordpress.com/.
